# Correction: A ternary system of meloxicam with matching hydrophilic polymer and cyclodextrin for improved stability in liquid preprations

**DOI:** 10.1039/d4ra90084g

**Published:** 2024-08-09

**Authors:** Si Li, Longfa Kou, Yimeng Qin, Yimeng Wang, Yinghua Sun, Zhonggui He, Xiaohong Liu

**Affiliations:** a School of Pharmacy, Shenyang Pharmaceutical University 103 Wenhua Road Shenyang 110016 China; b Wuya College of Innovation, Shenyang Pharmaceutical University 103 Wenhua Road Shenyang 110016 China sunyinghua77@aliyun.com +86-24-23986321 +86-24-23986321; c Department of Pharmacy, The Second Affiliated Hospital and Yuying Children's Hospital of Wenzhou Medical University Wenzhou 325035 China

## Abstract

Correction for ‘A ternary system of meloxicam with matching hydrophilic polymer and cyclodextrin for improved stability in liquid preprations’ by Si Li *et al.*, *RSC Adv.*, 2024, **14**, 21260–21268, https://doi.org/10.1039/D4RA02811B.

The authors regret that there were errors in the Author contributions and Acknowledgements sections of the published article. The corrected sections should read as follows.

**Author contributions**

All authors contributed to the study conception and design. Material preparation, data collection and analysis were performed by Si Li, Longfa Kou and Yimeng Qin. The first draft of the manuscript was written by Si Li and all authors commented on previous versions of the manuscript. All authors read and approved the final manuscript.

**Acknowledgements**

The authors acknowledge the work was supported by a research group from Shenyang Pharmaceutical University. This work was financially supported by National Natural Science Foundation of China (No. 82173766).

The Royal Society of Chemistry apologises for these errors and any consequent inconvenience to authors and readers.

